# Cytological Observations and Bulked-Segregant Analysis Coupled Global Genome Sequencing Reveal Two Genes Associated with Pollen Fertility in Tetraploid Rice

**DOI:** 10.3390/ijms22020841

**Published:** 2021-01-15

**Authors:** Nabieu Kamara, Yamin Jiao, Zijun Lu, Kelvin Dodzi Aloryi, Jinwen Wu, Xiangdong Liu, Muhammad Qasim Shahid

**Affiliations:** 1 State Key Laboratory for Conservation and Utilization of Subtropical Agro-Bioresources, South China Agricultural University, Guangzhou 510642, China; kamara.nabieu@yahoo.com (N.K.); 15515307073@163.com (Y.J.); zjlu@stu.scau.edu.cn (Z.L.); aloryikelvin@gmail.com (K.D.A.); jwwu@scau.edu.cn (J.W.); 2 Guangdong Provincial Key Laboratory of Plant Molecular Breeding, South China Agricultural University, Guangzhou 510642, China; 3 College of Agriculture, South China Agricultural University, Guangzhou 510642, China; 4 Guangdong Laboratory for Lingnan Modern Agriculture, South China Agricultural University, Guangzhou 510642, China

**Keywords:** BSA-seq, polyploid rice, neo-tetraploid, meiosis, pollen sterility

## Abstract

Neo-tetraploid rice with high fertility is a useful germplasm for polyploid rice breeding, which was developed from the crossing of different autotetraploid rice lines. However, little information is available on the molecular mechanism underlying the fertility of neo-tetraploid rice. Here, two contrasting populations of tetraploid rice, including one with high fertility (hereafter referred to as JG) and another with low fertility (hereafter referred to as JD), were generated by crossing Huaduo 3 (H3), a high fertility neo-tetraploid rice that was developed by crossing Jackson-4x with 96025-4x, and Huajingxian74-4x (T452), a low fertility autotetraploid rice parent. Cytological, global genome sequencing-based bulked-segregant (BSA-seq) and CRISPR/Cas9 technology were employed to study the genes associated with pollen fertility in neo-tetraploid rice. The embryo sacs of JG and JD lines were normal; however, pollen fertility was low in JD, which led to scarce fertilization and low seed setting. Cytological observations displayed low pollen fertility (25.1%) and approximately 31.3 and 27.2% chromosome lagging at metaphase I and II, and 28.8 and 24.8% chromosome straggling at anaphase I and II in JD, respectively. BSA-seq of F_2–3_ generations and RNA-seq of F_4_ generation detected a common fragment, i.e., 18,915,234–19,500,000, at chromosome 7, which was comprised of 78 genes associated with fertility. Among 78 genes, 9 genes had been known to be involved in meiosis and pollen development. Two mutants *ny1* (*LOC_Os07g32406*) and *ny2* (*LOC_Os07g32040*) were generated by CRISPR/Cas9 knockout in neo-tetraploid rice, and which exhibited low pollen fertility and abnormal chromosome behavior. Our study revealed that two unknown genes, *LOC_Os07g32406* (*NY1*) and *LOC_Os07g32040* (*NY2*) play an important role in pollen development of neo-tetraploid rice and provides a new perspective about the genetic mechanisms of fertility in polyploid rice.

## 1. Introduction

Polyploid species are widely found in plants and had higher economic value and confers greater stress resistance than diploid plants [1,2,3]. Polyploids showed stronger short-term adaptive potential during environmental change due to changes in gene expression and increased genetic diversity [3,4,5]. Autotetraploid rice was derived from diploid rice by chromosome doubling through colchicine treatment to enrich the breeding germplasm of rice, which showed great biological advantages, stress resistance and high heterosis [6,7,8,9,10]. However, low seed set is a major obstacle in the utilization of autotetraploid rice at commercial level [11,12,13,14].

Recently, two important tetraploid rice germplasms with high seed set and high pollen fertility, named as PMeS and Neo-tetraploid rice, have been developed [9,15]. These two fertile tetraploid germplasms were derived from the progenies of crosses between two intersubspecific autotetraploid rice lines. Up to now, six neo-tetraploid rice lines, including Huaduo1 to Huaduo5 and Huaduo8, have been reported by our research team [9,16,17,18]. These neo-tetraploid lines could overcome autotetraploid rice sterility and produce strong heterosis [9,16,17].

Autotetraploid lines had complicated reproductive defects, including abnormal embryo sac and pollen development, double fertilization, embryogenesis as well as endosperm development. Among these defects, high frequency of abnormal chromosome behaviors in pollen mother cells (PMC) during meiosis is one of the most common defects in autotetraploid rice [6,19]. In particular, the straggled chromosome was a main meiotic defect that led to autotetraploid sterility, including chromosome straggling at metaphase I and metaphase II, and chromosome lagging at anaphase I and anaphase II [6,19]. In contrast, neo-tetraploid lines do not show abnormal chromosomal behavior during meiosis, and hundreds of genes were differentially expressed in meiotic anthers of neo-tetraploid lines relative to autotetraploid lines [9,17,20,21]. Among these genes, the meiotic gene *OsMND1*, whose homolog plays a meiotic role in yeast and *Arabidopsis*, could partially improve the seed setting of autotetraploid line Balilla-4x by maintaining the balance of synapsis and recombination [22]. Loss of function of *MOF1* increased the percentage of straggled chromosomes in neo-tetraploid line Huaduo1 [22]. However, the molecular foundation of different meiotic process between neo-tetraploids and autotetraploids remained largely unknown.

The strategy of bulked segregant analysis (BSA) proposed by Michelmore et al. [23], provides a simple and more effective approach to rapidly identify candidate genomic regions that underlie the target genes based on whole genome resequencing of bulked populations and both parents [24]. Bulked-segregant analysis coupled with global sequencing (BSA-seq) has been successfully applied to clone genes in plants, including rice [24,25,26,27,28], melon [29], soybean [30,31], tomato [32], chickpea [33,34], cucumber [35], maize [36], *Brassica rapa* [37], and strawberry [38]. Four sets of chromosomes in autotetraploid and neo-tetraploid rice made it difficult to map genes associated with fertility. BSA-seq is expected to the shorten period of gene identification in neo-tetraploid rice, but its application has not been demonstrated in tetraploid rice.

In the present study, we constructed F_2_, F_3_ and F_4_ populations derived from a cross between a neo-tetraploid line, Huaduo3 (H3), and an autotetraploid line, Huajingxian74-4x (T452). BSA-seq based on F_2_, F_3_ and F_4_ populations was employed to map the meiosis related region. Moreover, we identified deferentially expressed genes between high fertility lines (H3 and JG) and low fertility lines (T452 and JD) from the linkage region. Then, CRISRP/Cas9 was employed to edit the candidate genes in the neo-tetraploid line Huaduo1 (H1). As expected, the mutant lines displayed low seed set, low pollen fertility and high frequency of straggled chromosomes during meiosis. These results reveal two important meiotic genes in neo-tetraploid rice and also provide a foundation for understanding the molecular mechanism for different meiosis processes between neo-tetraploid and autotetraploid rice.

## 2. Results

### 2.1. Morphological and Cytological Observations of Bulks (JG and JD) with Different Fertility

To study the molecular foundation of the meiosis process between neo-tetraploid and autotetraploid, two extreme bulks, with high seed set and low seed set, were developed from a cross between a neo-tetraploid line, Huaduo3 (H3), and an autotetraploid line, Huajingxian74-4x (T452) (Figure S1). The high seed set bulk was named as JG, which consisted of 40 F_2_ plants with more than 80% seed set. The low seed set bulk was named as JD, which also contained 40 F_2_ plants with less than 30% seed set. JG and JD were self-crossed to generate F_3_ and F_4_ generations, while plants with less than 80% seed set in JG and more than 30% in JD were removed from both generations.

**Figure 1:**
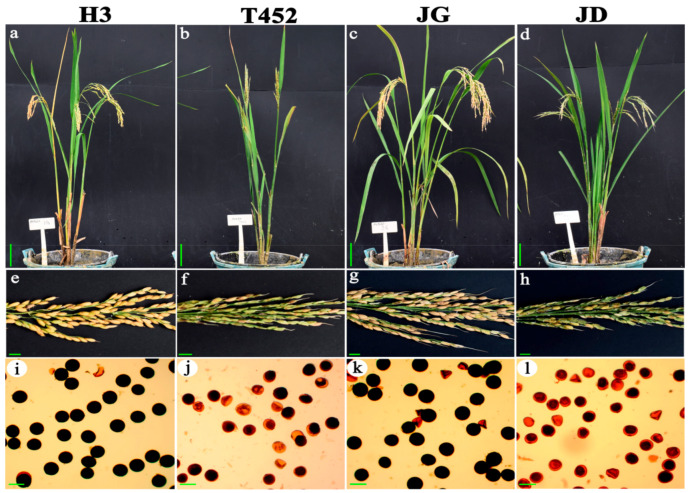
Phenotypic characterization of hybrid lines and their parents. (**a**–**d**) Plant morphology of H3, T452, JG and JD at mature stage. Bars = 10 cm. (**e**–**h**) Mature panicles of H3, T452, JG and JD (from left to right). Bars = 1 cm. (**i**–**l**) Pollen fertility of H3, T452, JG and JD. Bars = 50 μm.

JD and T452 displayed significantly lower seed set and pollen fertility than JG and H3 (Figure 1). The seed set of H3, T452, JG and JD were 80.43%, 15.30%, 82.52% and 18.84%, respectively (Table 1, Figure 1). There was no obvious difference in embryo sac fertility among four materials (Table 1). The normal embryo sac frequencies of the JG, JD, H3 and T452 were 93.52%, 85.03%, 91.99%, and 85.24%, respectively. However, abundant stained abortive or typical abortive pollens were found in JD and T452 (Figure 1, Table S1). Pollen fertility of JD (25.08%) and T452 (23.70%) was significantly lower than JG (88.53%) and H3 (87.45%) (Table 1).

Moreover, whole-mount eosin B-staining confocal laser scanning microscopy (WE-CLSM) analysis was also employed to observe the embryogenesis and endosperm development at 3 days after flowering (DAF). In this stage, the primary endosperm nucleus began to enrich the embryo sac, expanding from the apical end to the basal (or chalaza) end and begins to form the endosperm wall, and the embryo starts to undergo cellular differentiation (Figure S2). Some abnormalities were found in these stages, including a small globular-shaped embryo sac, embryo sac degeneration, abnormal position of polar nuclei, multiple polar nuclei in the embryo sac, delayed embryo development, embryo and endosperm abnormality, embryo fertilization stagnates, an unfertilized embryo sac, and a single fertilized egg cell. The normal frequencies of fertilized samples of H3, T452, JG, and JD were 81.97%, 10.96%, 81.90% and 24.86%, respectively (Table 1). These results indicated that T452 and JD displayed defects during double fertilization and embryogenesis.

### 2.2. Comparison of Chromosomal Behavior between H3, T452, JG and JD

**Figure 2:**
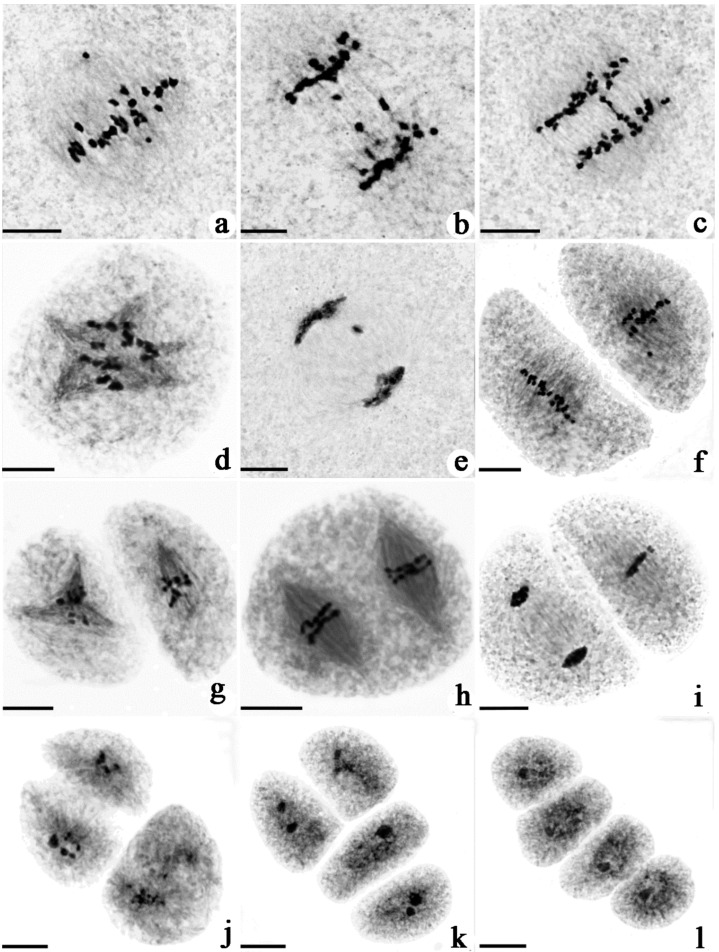
Abnormal chromosome behavior during pollen mother cells meiosis in JD. (**a**) Chromosome lagging at metaphase I; (**b**,**c**) chromosome straggling at anaphase I; (**d**) multipolar spindle at metaphase I; (**e**) micronuclei at telophase I; (**f**) chromosome lagging at metaphase II; (**g**–**i**) abnormal cell shape and asynchronous division of chromosomes during meiosis II; (**j**) abnormal triad; (**k**) abnormal “T” shape tetrad; (**l**) abnormal line shape tetrad. Bars = 10 μm.

To understand the cytological difference between tetraploid rice with different fertility levels, the meiotic chromosomal behavior of PMCs was observed. The meiotic process was similar in H3, T452, JG and JD, which could be divided into nine stages, including prophase I (leptotene, zygotene, pachytene, diplotene, diakinesis), metaphase I, anaphase I, telophase I, prophase II, metaphase II, anaphase II, telophase II, and tetrad (Figure S3). Some chromosomal behavior abnormalities were found in these stages, including chromosome lagging at metaphase, chromosome straggling at anaphase, micronucleus, spindle abnormality, abnormal cell shape and asynchronous division, and abnormal triad shape (Figure 2). Four key meiotic stages—metaphase I, anaphase I, metaphase II, and anaphase II—were selected to summarize the frequency of abnormalities. JG had less abnormal PMCs compared with JD. The frequency of PMCs with abnormal chromosome behavior in JD at metaphase I, anaphase I, metaphase II, and anaphase II were 31.27%, 28.76%, 27.23% and 24.82%, while JG only displayed 16.56%, 8.36%, 2.42% and 2.07% PMCs with abnormalities (Table 1). The above results indicate that the high frequency of abnormal chromosome behavior during PMC meiosis was an important reason for the difference in pollen fertility between JG and JD.

### 2.3. High Fertility Blocks Identification by BSA-seq and BSR-seq Using JG Bulk, JD Bulk and Their Parents

We obtained high-quality sequencing datasets with high genome coverage and average depth by BSA-seq of F_2_ generation (Table S2). A total of 11.68 Gb, 11.49 Gb, 33.15 Gb and 33.71 Gb clean data were obtained from H3, T452, JG bulk and JD bulk, respectively. The regions of the four samples mapped onto the reference genome were above 97.80%, the average coverage depths were 23–25 for parents and 73–74 for bulks, and the genome coverage was above 92.11% (Table S2). In total, 3,159,637 single nucleotide polymorphisms (SNPs) and insertions and deletions (InDels) polymorphic loci were identified between H3 and T452, while 2,553,364 polymorphic loci were identified between JG bulk and JD bulk. In F_2_, a total of 1,346,124 high-quality SNPs were obtained for ΔSNP-index analysis. Correlation analysis mapped one quantitative trait locus (QTL) (ΔSNP-index > 0.09) on chromosome 7 (18,180,000–19,500,000).

BSA-seq of F_3_ generation also revealed high-quality sequencing datasets as produced by F_2_ generation (Table S3). The regions of the four samples mapped onto the reference genome were above 98.01%, the average coverage depths were 28–34 for parents and 67 for bulks, and the genome coverage was above 91.86% (Table S3). In total, 3,159,637 polymorphic loci (SNPs and InDels) were identified between H3 and T452, while 2,553,364 polymorphic loci were identified between JG bulk and JD bulk. In F_3_, a total of 1,343,958 high-quality SNPs were obtained for ΔSNP-index analysis. Correlation analysis mapped three QTLs (ΔSNP-index > 0.14), including two on chromosome 1 (33,900,000–33,900,000, 33,920,000–33,940,000) and one on chromosome 7 (18,450,000–22,140,000).

**Figure 3:**
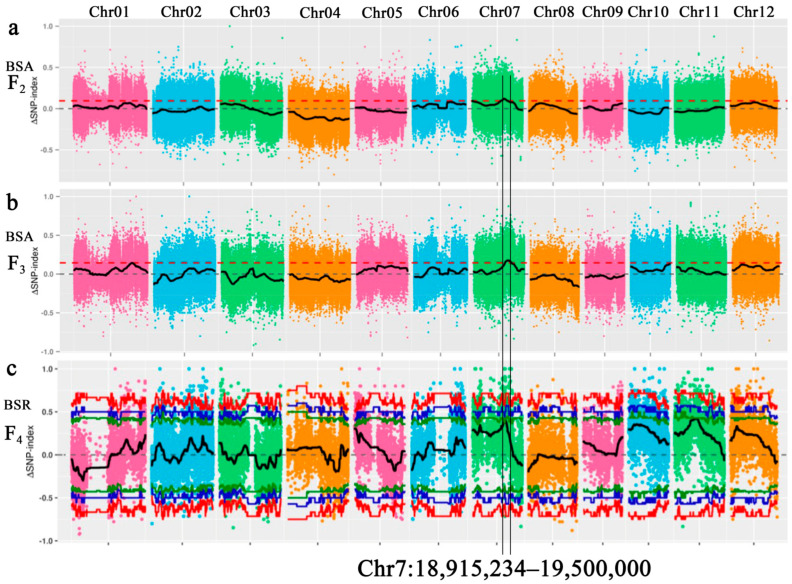
Identification of the hot-region for high fertility through the single nucleotide polymorphism SNP-index association analysis method. The *X*-axis represents the position of 12 chromosomes of rice and the *Y*-axis represents the Δ (SNP-index). The color dots represent the Δ (SNP-index) value of every SNP locus. The black lines show the Δ (SNP-index) value of fitting results. (**a**): The Δ (SNP-index) graph of F_2_ generation. (**b**): The Δ (SNP-index) graph of F_3_ generation. (**c**): The Δ (SNP-index) graph of F_4_ generation. The red dotted line shows the association threshold.

A total of 6.37 Gb, 6.26 Gb, 45.97 Gb and 31.33 Gb clean data were obtained by bulked segregant RNA-seq (BSR-seq) of F_4_ generation from H3, T452, JG bulk and JD bulk, respectively. The Q30 base percentage was above 94.02% and the GC content in each sample sequencing data was between 55 and 57% (Table S4). In this generation, 65,202 SNPs were obtained for ΔSNP-index correlation analysis, which mapped two QTLs (ΔSNP-index > 0.90) on chromosome 7 (18,915,234–19,692,348, 19,736,756–19,748,279). Together, the results of BSA-seq and BSR-seq showed that only one high fertility QTL was mapped to the segment 18,915,234–19,500,000 on chromosome 7 (Figure 3).

### 2.4. Expression Analysis of Candidate Genes in High Fertility Blocks

**Figure 4:**
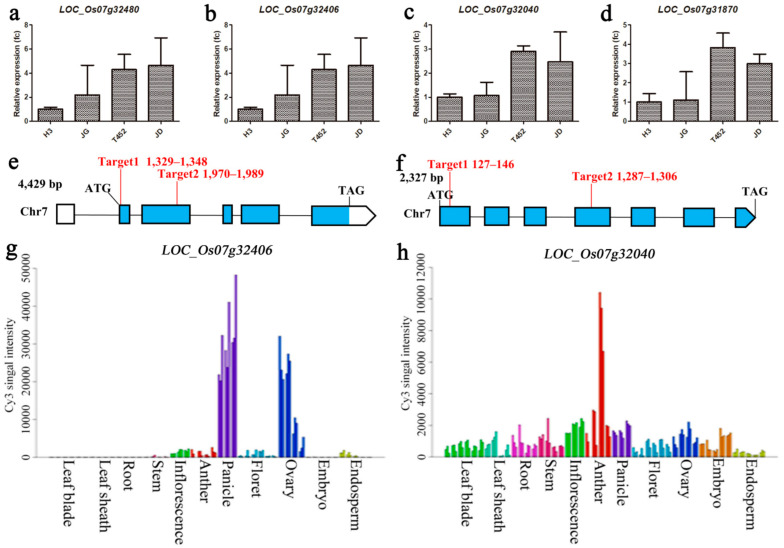
Expression analysis of 4 candidate genes in high fertility blocks. QRT-PCR results of *LOC_Os07g32480* (**a**), *LOC_Os07g32406* (**b**), *LOC_Os07g32040* (**c**), and *LOC_Os07g31870* (**d**). The ubiquitin gene was used as an internal control. Fc, fold change. The error bars (**a**–**d**) indicate SE. The gene structure and target site for CRISPR/Cas9 of *LOC_Os07g32406* (*NY1*) (**e**) and *LOC_Os07g32040* (*NY2*) (**f**). The predicted expression patterns of *LOC_Os07g32406* (*NY1*) (**g**) and *LOC_Os07g32040* (*NY2*) (**h**).

We detected nine genes in high fertility QTL, which were related to pollen mother cell meiosis and pollen fertility, and these nine genes were *LOC_Os07g31870*, *LOC_Os07g32010*, *LOC_Os07g32020*, *LOC_Os07g32040*, *LOC_Os07g32170*, *LOC_Os07g32406*, *LOC_Os07g32480*, *LOC_Os07g32650* and *LOC_Os07g32660*. To confirm their expression levels in JG, JD and their parents, all nine genes were selected for qRT-PCR analysis at the meiosis stage. *LOC_Os07g32480*, *LOC_Os07g32406*, *LOC_Os07g32040* and *LOC_Os07g31870* showed down-regulation in high fertility materials (H3 and JG) relative to low fertility materials (T452 and JD) (Figure 4a–d).

According to the Rice Genome Annotation Project Database [39], *LOC_Os07g32406* is comprised of 4429 base pairs (bp) with six exons and five introns (Figure 4e). The coding sequence consists of 1770 bp and it encodes a protein with 589 amino acids (aa). The *LOC_Os07g32406* is designated as *NY1* in this study. In gene expression profile analysis by RiceXPro [40], *NY1* show high expression level in panicle and ovary (Figure 4g), suggesting that *NY1* may play an important role in reproduction.

*LOC_Os07g32040* is comprised of 2327 bp with seven exons and six introns (Figure 4f). The coding sequence contained 1311 bp and it encodes a eukaryotic translation initiation factor 3 sub-unit E with 426 aa. The *LOC_Os07g32040* is designated as *NY2* in this study. *NY2* was preferentially expressed during another development (Figure 4h), suggesting that *NY2* play an important role in male reproduction.

### 2.5. Knockout of Two Candidate Genes Causes Pollen Abortion in Neo-Tetraploid Rice

In order to explore the reproductive roles of *NY1* and *NY2*, we generated knockout mutants of *NY1* and *NY2* in the neo-tetraploid line Huaduo1 by using the CRISPR/Cas9 genome editing system [41]. We obtained at least 34 independently regenerated transgenic plants of *NY1* and *NY2* after the transformation (Tables S5 and S6). The mutant lines were grown in the field, and the T1 and T2 mutant plants were sequenced (Tables S5 and S6). We further selected the homozygous mutants of Ny1 and Ny2 to observe the pollen fertility and pollen development process.

**Figure 5:**
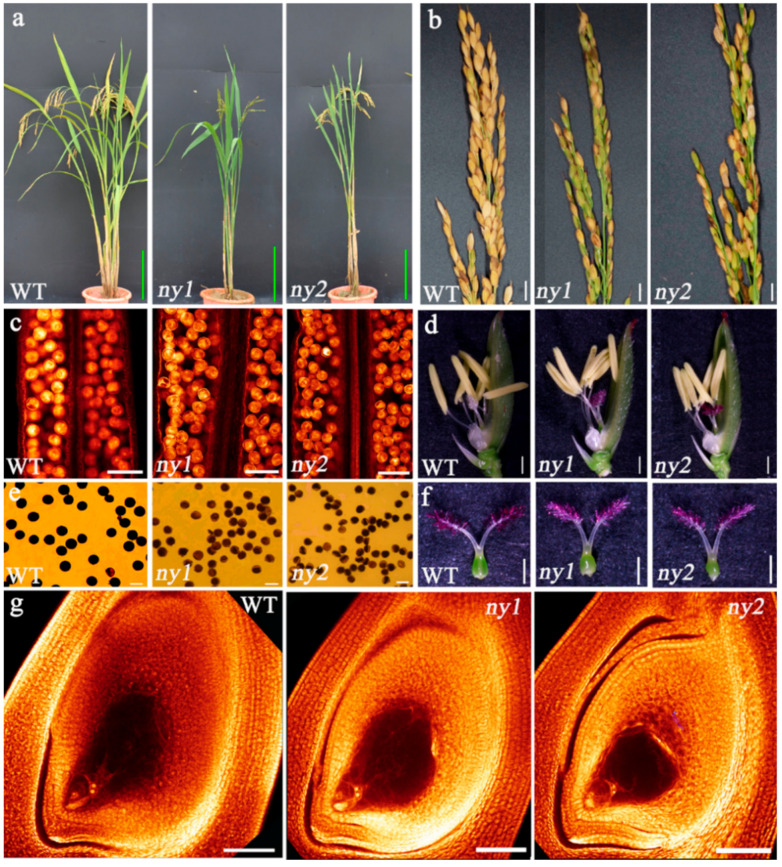
Phenotypic characterization of wild type (WT), *ny1* and *ny2* mutant lines. (**a**) Plant morphology of the WT, *ny1* and *ny2* mutants at mature stage. Bars = 20 cm. (**b**) Mature panicles. Bars = 1 cm. (**c**) Pollen grains observed by whole-mount eosin B-staining confocal laser scanning microscopy (WE-CLSM). Bars = 100 μm. (**d**) Florets. Bars = 1 mm. (**e**) I_2_-KI staining of pollen grains. Bars = 50 μm. (**f**) Pistils. Bars = 1 mm. (**g**) Mature embryo sac. Bars = 100 μm.

The *ny1* and *ny2* (mutant lines) showed normal plant types as well as normal vegetative development (Figure 5a). Phenotypic analysis showed that there were remarkable differences in plant growth or panicle morphology between the mutant lines and the wild type plants (Figure 5). Plant height, panicle length, panicle number, total grain number, and 1000-grain weight were significantly reduced in all mutants compared to their wild-type plants. Grain length and grain width of the mutants were similar to that of wild-type plants. Interestingly, the panicles of *ny1* and *ny2* showed many unfilled grains, and the seed setting rate of *ny1* (43.34%) and *ny2* (49.48%) were significantly lower than wild type plant (78.00%). As a consequence, grain yield in the mutants were markedly reduced compared to the wild plants (Table 2). These results revealed potential reproductive defects in *ny1* and *ny2*. The floral developments of *ny1* and *ny2* were normal (Figure 5d,f), and mature embryo sac fertility of *ny1*, *ny2* and wild type (WT) were higher than 85% (Figure 5g, Table 2), but the pollen fertility of *ny1* (38.66%) and *ny2* (26.65%) was significantly lower than the pollen fertility of WT (93.05%) (Figure 5c,e, Table 2). These results suggested that the low seed setting in *ny1* and *ny2* was associated with pollen development.

### 2.6. Analysis of Chromosome Behavior in ny1, ny2 and WT

**Figure 6:**
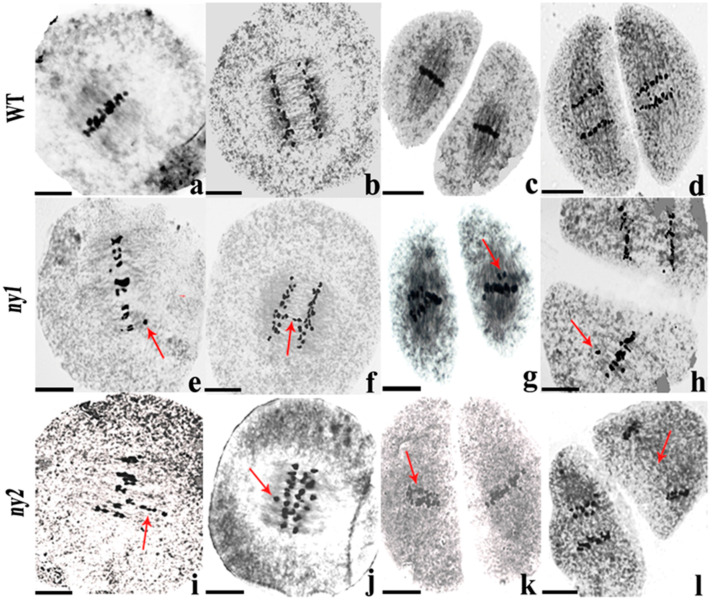
Chromosome behavior of PMC meiosis in wild type (WT), *ny1* and *ny2* mutant lines. (**a**–**d**) indicate metaphase I; anaphase I; metaphase II; anaphase II images of wild-type. (**e**–**h**) are showing metaphase I; anaphase I; metaphase II; anaphase II images of *ny1* and (**i**–**l**) represent metaphase I; anaphase I; metaphase II; anaphase II images of *ny2*. Red arrows indicate abnormal chromosomes. Bars = 50 μm.

To study the effects of the *NY1* and *NY2* genes on plant reproduction, we compared chromosome behavior of meiotic pollen mother cells between *ny1*, *ny2* and WT. We detected few abnormalities in WT plants. However, there were remarkable differences in meiosis process of *ny1* and *ny2* compared to WT plants. At metaphase I, 64.71% of 561 observed *ny1* PMCs and 65.97% of 238 observed *ny2* PMCs contained straggling chromosomes, while only 14.66% was found in WT (688). At anaphase I, 66.48% of 179 observed *ny1* PMCs and 62.37% of 110 observed *ny2* PMCs displayed chromosome lagging, while only 15.83% was found in WT (139). At metaphase II, 71.25% and 90.24% PMCs exhibited chromosome straggling in *ny1* and *ny2*, while only 15.44% was found in WT (149). At anaphase II, 71.69% and 87.23% cells showed chromosome lagging in *ny1* and *ny2*, while 51.35% was found in WT (37). The frequency of PMCs with chromosome behavior abnormalities in *ny1* or *ny2* were both higher than WT at these observed stages (Table 3, Figure 6). These results indicate that both *NY1* and *NY2* affect chromosome behavior during meiosis in neo-tetraploid rice.

## 3. Discussion

### 3.1. BSA-Seq is an Effective Technique to Identify Genes in Polyploid Plants

BSA-seq is an easy and effective tool to map QTLs associated with complex traits in plants. For example, 3.29 Mb candidate region was mapped in tomato by BSA-seq and five kompetitive allele-specific PCR KASP markers were employed to fine map a gene, *Solyc01g007130*, associated with leaf mold [32]. In maize, *VKS1* was cloned by BSA-seq, which regulates mitosis and cytokinesis during endosperm development [36]. BSA-seq also accelerated the breeding process of rice cultivar with salt-tolerance [24].

Different types of populations were used for cloning genes in diploid plants, including common F_2_ [24,29,31,32,33,35], BC_6_F_2_ [36], BC_4_F_2_ [37]. In general, the F_2_ population is easier to develop and shorten the mapping duration than other populations. However, the ΔSNP-index peak was only higher than 0.09 in our F_2_ population, which could not distinguish potential QTL from other unlinked regions. Then, we further developed the F_3_ and F_4_ generations to perform BSA-seq and interesting results were obtained. The ΔSNP-index peaks were higher than 0.14 and 0.90 in F_3_ and F_4_ generations, respectively. These results suggest that F_3_ and F_4_ generation populations will be better than common the F_2_ population to obtain good results in polyploid plants. Similarly, the 2.45 Mb region associated with flower color was identified in *Brassica juncea* (allopolyploid) by using BC_4_ [42]. It is difficult to identify homozygous individuals from the F_2_ population in autotetraploid plants because of their four groups of homologous chromosomes.

BSA-seq generally maps the QTL at a Mb-level interval, which contains numbers of genes. Fine mapping is still required to be performed by using simple sequence repeat SSR markers [42], KASP markers [32], cleaved amplified polymorphic sequence CAPS markers [32] or specific length amplified fragment sequence SLAF-seq [35]. In addition, combining with expression analysis, such as qRT-PCR, RNA-seq is also helpful for further screening the candidate genes [33,35]. In this study, we combined three generation BSA-seq results to map QTL associated with fertility in a 0.59-Mb candidate region on chromosome 7. We further identified four candidate genes by qRT-PCR from this QTL and two genes were verified to reveal their reproductive roles in meiotic anthers by CRISPR/Cas9. These results suggested that BSA-seq of F_3_ and F_4_ progenies have definite advantage to identify fertility related genes in polyploid plants.

### 3.2. NY1 and NY2 Play Crucial Role in Pollen Mother Cell Meiosis

Meiosis process has a great effect on plant reproductive development, and chromosome behavior and configuration play an important role in pollen development [43,44]. A number of genes that the regulate meiosis process have been identified, such as *OsREC8*, *OsSGO1*, *ZIP4*, *HEI10*, *MRE11*, *OsDMC1*, *OsMND1* and *MOF1a*. Mutants of these meiotic genes caused many chromosome abnormalities during the meiosis process because of abnormal homologous synapsis or double strand break [21,22,45,46,47,48,49,50]. The abnormal meiosis process is one of the most important factors that cause sterility in autotetraploid rice [6,17,51,52,53,54,55]. Here, low fertility lines (JD and T452) showed higher frequency of abnormal chromosome behavior than high fertility lines (JG and H3). The main chromosomal abnormalities include chromosome lagging, chromosome straggling, micronucleus, spindle abnormality, abnormal cell shape and asynchronous division, and abnormal triad shape. These results are consistent with previous studies, who also detected different types of chromosomal abnormalities in low fertility polyploid rice [6,17,18,51,52,53,54,55]

Many research studies have focused on polyploid meiotic defects. *ASY1* and *ASY3* are associated with meiotic stability and their derived alleles improve meiotic traits in autotetraploid *Arabidopsis arenosa*. From diploid to autotetraploid or from autotetraploid (low fertility) to neo-tetraploid (high fertility) rice, thousands of genes differentially expressed in meiotic anthers, including many important meiotic genes and tapetal genes like *RAD51*, *SMC2*, *OsABCG26*, *PTC1*, *SSP* and *DPW* [6,9,17,18,19,20,21,56]. Many of these differentially expressed genes (DEGs) were identified as meiosis-related genes, but still functional verifications of most of DEGs are unknown [57,58]. Recently, *OsMND1* and *MOF1* were found to be associated with meiotic stability in tetraploid rice [21,22]. However, the knowledge about genes/QTLs that regulates meiotic abnormalities in autotetraploid rice is still limited.

Here, we used BSA-seq to map a QTL on Chr7 that associated with meiosis development of tetraploid rice and found four differentially expressed genes in this region. Among these four genes, *BRK1* (*LOC_Os07g32480*) encodes a Bub1-Related Kinase, which is essential for generating proper tension between homologous kinetochores at meiotic metaphase I of rice [59]. Moreover, we produced knock-out mutants of *NY1* and *NY2*, which preferentially expressed in reproductive tissues, by the CRISPR/Cas9 system. Both *ny1* and *ny2* mutants exhibited significantly low pollen fertility compared to WT plants (neo-tetraploid rice). Loss of function of *NY1* or *NY2* increased the frequency of PMCs with abnormal chromosomes during the meiosis process, which was consistent with the meiosis process in JD (low fertility bulk). We conferred that the QTL is important to meiotic stability of tetraploid rice and consists of important essential meiotic genes. Our findings provide a foundation to understand the molecular mechanism of meiotic stability in tetraploid rice.

## 4. Materials and Methods

### 4.1. Plant Material

A high fertility neo-tetraploid rice line, Huaduo3 (H3), which was developed by crossing Jackson-4x with 96025-4x, was used to generate the F_1_ hybrid by crossing with a low fertility autotetraploid rice line, Huajingxian74-4x (T452) that was developed from a diploid rice cultivar (Huajingxian74-2x). F_2_ to F_4_ populations were obtained from self-fertilization progenies of F_1_ hybrid. Those plants with more than 80% seed setting were selected to construct JG bulk, while those plants with less than 30% seed set rate were selected to construct JD bulk in each generation. Another neo-tetraploid line, Huaduo1, was used as the receptor during CRISPR/Cas9 transgenesis to generate *ny1* and *ny2* mutants. All the materials were planted at the experimental farm of South China Agricultural University (SCAU) under natural conditions, and management practices followed the recommendations for the area.

### 4.2. Cytological Observation

The inflorescences were collected from rice plants with 0 to 4 cm between their flag leaf cushion and the second-to-last leaf cushion, fixed in Carnoy solution (ethanol: acetic acid, 3:1 *v*/*v*) for more than 24 h, and then stored in 70% ethanol. Chromosome behavior and configuration were observed and analyzed as described by Chen et al. [17]. Meiosis stages were classified and explained according to He et al. [54]**.**

The pollen fertility was observed according to Ghouri et al. [60]. The mature pollen grains were observed by staining with 1% I_2_-KI under a microscope (Motic BA200, Lampertheim, Germany).

A whole-mount eosin B-staining confocal laser scanning microscopy (Leica Microsystems, Heidelberg, Germany) was used to investigate the mature embryo sac fertility according to Li et al. [13] with minor modifications. The spikelets of pre-flowering were collected and fixed in a Formaldehyde–Acetic acid–Ethanol solution (70% ethanol: acetic acid: formaldehyde = 18:1:1, *v*/*v*) for at least 24 h. Then, the samples were stored in 70% alcohol at 4 °C. The isolated ovaries were hydrated consecutively in 50%, 30%, and 10% ethanol and distilled water for 30 min, respectively. After an eosin B (10 mg/L in 4% sucrose solution) staining procedure for 10 h, the samples were dehydrated sequentially in 10%, 30%, 50%, 70%, 90%, and 100% (three times) alcohol for 30 min, respectively. The dehydrated samples were kept in a methyl salicylate and ethanol solution (1:1, *v*/*v*) for 1 h. Finally, the samples were stored in pure methyl salicylate for 1 h and observed under the Leica SPE laser scanning confocal microscope.

### 4.3. qRT-PCR Analysis of Candidate Gene Expression

A total of nine genes were selected for validation by qRT-PCR. The gene-specific primers (Table S7) were designed using Primer Premier 5.0 software. The total RNA was extracted by AG RNAex Pro Reagent and was reverse-transcribed into cDNA with the Evo M-MLV RT Kit of Accurate Biotechnology (Hunan, China) Co., Ltd. All operations were implemented according to the Kit instructions. The qRT-PCR reaction procedure was performed on the Lightcycler480 system (Roche, Basel, Switzerland) as follows: 30 s at 95 °C, 40 cycles of 95 °C denaturation for 10 s and 60 °C annealing and extension for 30 s. The genes relative expression levels were calculated using the 2^−ΔΔCt^ method [61]. All qRT-PCR reactions were performed in triplicate.

### 4.4. Mixed Pool Construction and Analysis of Mixed Pool Sequencing Data

Young leaves of H3, T452, F_2_ plants and F_3_ plants were collected and were stored at −80 °C. The leaves of 40 F_2_ plants with high fertility > 80% were mixed to develop JG bulk, while leaves of 40 F_2_ plants with low fertility < 30% were mixed to develop JD bulk. The same work was repeated in F_3_ generation. The DNA from leaves of two parents and two bulks of F_2_ and F_3_ generations were extracted, and their libraries constructed and sequenced based on the manufacturer’s instructions Illumina HiSeq [62].

In the F_4_ generation, the meiotic anthers (identified based on spikelet length) from H3, T452, and F_4_ plants were collected. A total of 90 anthers were independently extracted from each plant, quick-frozen in liquid nitrogen, and stored at −80 °C. Similar to F_2_ and F_3_ generation, the F_4_ plants with high (>80%) and low fertility (<30%) were designated as JG and JD bulk, respectively. The anthers from 20 plants of each H3, T452, JG, and JD were mixed to construct a pool. The mixed anther samples were sent to Biomark Biotechnology Co., Ltd. for RNA extraction, libraries construction and sequencing based on the manufacturer’s instructions Illumina HiSeq [62].

The quality of the original reads (paired-end sequences) obtained by sequencing were evaluated and low-quality reads were filtered. The obtained clean reads were used for subsequent bioinformatics analysis. Aligned clean reads were mapped onto the MSU7 reference genome [63]. The GATK software was used to identify SNPs and InDels [64], and the SnpEff software was used to annotate the SNPs and InDels [65]. SNP-index and InDel-index were calculated to identify the candidate regions associated with fertility [66].

### 4.5. Development and Identification of Mutant Plants in Huaduo1

CRISPR/Cas9 system was used to generate mutation of candidate genes as previously reported [41]. The two targets were designed for each candidate gene to obtain single guide RNA (sgRNA) expression cassettes (U6a and U6b promoters), which were incorporated into the CRISPR/Cas9 vector pLYCRISPR/Cas9Pubi-H. Then, the vectors were transferred into Huaduo1. The target region for each mutant was amplified by PCR, and the segment was subjected to Sanger sequencing. The T_2_ plants of homozygous mutant were used for phenotypic and genotypic analyses. The sequences of these primers are listed in Table S7.

### 4.6. Investigation of Agronomic Traits and Data Analysis

Agronomic traits, including plant height, days to 50% flowering, Flag leaf length, Flag leaf width, number of panicles per plant, grain length, grain width, grain length to width ratio, 1000-grain weight, filled grains per plant, total grains per plant, grain yield per plant and seed setting, were investigated. The standard for investigating these agronomic traits was according to the protocols of People’s Republic of China for the registration of a new plant variety Distinctness, Uniformity and Stability (DUS) test guidelines of rice (Guidelines for the DUS test in China, 2012) [9]. A one-way analysis of variance (ANOVA) and Duncan’s multiple range test (DMRT) was used to identify significant (*p* < 0.05) differences between group averages, using the SPSS 19.0 statistical software.

## 5. Conclusions

In this study, BSA-seq of F_2–3_ generations, and RNA-seq of F_4_ detected a common fragment on chromosome No.7, which is comprised of 78 genes associated with fertility. Among these 78 genes, 9 genes had been known to be involved in meiosis and pollen development. Furthermore, we systematically investigated the embryo sac fertility, pollen fertility and chromosome behavior of JG and JD, and embryo sac fertility of JG and JD lines was found to be normal. However, pollen fertility was low in JD, which led to scarce fertilization and low seed setting. Moreover, Cytological observation displayed higher frequency of abnormal chromosome behaviors during meiosis in JD. Two mutants, *NY1* and *NY2*, associated with meiosis and pollen development were generated and the gene functions were validated using CRISPR/Cas9 gene knockout, and they showed low pollen fertility and abnormal meiosis. Overall, our results provide strong evidence that *NY1* and *NY2* play a critical role in pollen development of neo-tetraploid rice, which in turn is vital for maintaining rice seed setting. Our findings offer a foundation for understanding the molecular mechanism of meiotic stability in tetraploid rice and provide a new perspective to genetic research of fertility in polyploid rice.

## Figures and Tables

**Table 1:** Agronomic traits of JG bulk, JD bulk and their parents during early and late seasons in 2019.

Traits	H3	T452	JG	JD
Total grains/panicle	136.9 ± 13.4 B	116.0 ± 25.0 C	159.4 ± 22.2 D	120.7 ± 46.1 BC
Seed setting (%)	80.4 ± 3.0 A	15.3 ± 4.7 C	82.5 ± 4.2 A	18.8 ± 9.8 B
Pollen fertility (%)	87.5 ± 2.4 A	23.7 ± 6.8 B	88.5 ± 4.8 A	25.1 ± 5.1 B
Embryo sac fertility (%)	92.0	85.2	93.5	85.0
Embryo sac fertility at 3 DAF (%)	82.0	11.0	81.9	24.9
Chromosome lagging at metaphase I (%)	17.4 ± 1.7	13.9 ± 1.3	16.6 ± 2.2	31.3 ± 4.8
Chromosome straggling at anaphase I (%)	17.6 ± 0.3	13.6 ± 1.4	8.4 ± 0.6	28.8 ± 7.4
Chromosome lagging at metaphase II (%)	5.4 ± 0.3	3.1 ± 1.9	2.4 ± 1.4	27.2 ± 1.4
Chromosome straggling at anaphase II (%)	0.7 ± 0.7	0.0 ± 0.0	2.1 ± 1.3	24.8 ± 2.0

Least significant difference (LSD) was used in the multiple comparison tests for each trait. Different letters between two samples indicate significant differences (*p* value < 0.01). H3, T452, JG, JD and DAF indicate Huaduo3, Huajingxian74-4x, plants with high fertility, plants with low fertility and days after flowering, respectively.

**Table 2:** Mean performance of agronomic traits, pollen fertility and embryo sac fertility of WT, *ny1* and *ny2* mutant lines.

Traits	WT	*ny1*	*ny2*
Plant height (cm)	112.0 ± 6.3 a	102.2 ± 7.5 b	101.6 ± 6.9 b
Days to 50% flowering	101.0 ± 0.0 b	107.3 ± 1.5 a	109.7 ± 1.5 a
Flag leaf length (cm)	35.5 ± 2.6 a	29.4 ± 4.1 c	32.1 ± 3.8 b
Flag leaf width (cm)	2.3 ± 0.1 a	2.1 ± 0.3 b	2.2 ± 0.2a b
Number of panicles	6.0 ± 0.7 a	4.0 ± 1.0 b	4.5 ± 0.9 b
Grain length (mm)	9.4 ± 0.2 a	9.4 ± 0.7 a	9.2 ± 0.4 a
Grain width (mm)	3.5 ± 0.1 a	3.3 ± 0.2 b	3.4 ± 0.1 ab
Grain length to width ratio	2.7 ± 0.1 b	2.8 ± 0.3 a	2.7 ± 0.1 ab
Panicle length (cm)	27.1 ± 1.0 a	23.6 ± 2.3 b	23.9 ± 2.1 b
Total grains /plant	768.0 ± 126.1 a	293.6 ± 118.2 c	372.8 ± 108.1 b
Filled grains/plant	599.6 ± 94.4 a	128.4 ± 76.0 c	186.8 ± 72.3 b
1000-grain weight (g)	36.3 ± 1.4 a	26.5 ± 5.5 c	29.5 ± 2.8 b
Grain yield /plant (g)	21.6 ± 3.4 a	3.6 ± 2.4 c	5.5 ± 2.3 b
Seed setting (%)	78.0 ± 4.2 a	43.3 ± 14.2 b	49.5 ± 10.2 b
Pollen fertility (%)	93.1 ± 3.3 a	38.7 ± 36.3 b	26.7 ± 33.6 b
Embryo sac fertility (%)	90.1	85.1	87.2

Least significant difference (LSD) was used in the multiple comparison for each trait. Different lower-case letters between two samples indicated significant differences (*p* value < 0.05).

**Table 3:** Frequency of pollen mother cells with abnormal chromosome behavior during meiosis in WT, *ny1* and *ny2* mutant lines.

Stage	Wild Type (WT)		*ny1* (Mutant Line)		*ny2* (Mutant Line)	
	Number of Cells	Abnormal (%)	Number of Cells	Abnormal (%)	Number of Cells	Abnormal (%)
Metaphase I	688	14.7	561	64.7	238	66.0
Anaphase I	139	15.8	179	66.5	110	62.4
Metaphase II	149	15.4	80	71.3	82	90.2
Anaphase II	37	51.4	113	71.7	47	87.2

## Data Availability

Not applicable.

## Supplementary Materials

The following are available online at https://www.mdpi.com/1422-0067/22/2/841/s1, Figure S1: Breeding procedure of JG and JD inbred lines, Figure S2: Mature embryo sac of neo-tetraploid inbred lines and their parents and embryo sac at 3 days after flowering (DAF), Figure S3: Normal chromosome behavior during pollen mother cells meiosis in JG, Table S1: Pollen fertility of JG and JD hybrid lines and their parents: title, Table S2: Quality Assessment of Whole Genome Sequencing in F_2_ Generation Mixed Pool, Table S3: Quality Assessment of whole genome sequencing of mixed pool in F_3_ Generation, Table S4: Quality evaluation of transcriptome sequencing in F_4_ generation pool, Table S5: Mutation results of T_0_ knockout *ny1* and *ny2* by CRISPR/Cas9, Table S6: Mutation results of T_1_ knockout *ny1* and *ny2* by CRISPR/Cas9, Table S7: List of primers used in this study.

## Abbreviations

BSA	Bulked segregant analysis
DAF	Days after flowering
DGEs	Differentially expressed genes
qRT-PCR	Quantitative real-time polymerase chain reaction
PMCs	Pollen mother cells
QTL	Quantitative trait locus
WE-CLSM	Whole-mount eosin B-staining confocal laser scanning microscopy
WT	Wild type
