# Supplementary material for: Cytological Observations and Bulked-Segregant Analysis Coupled Global Genome Sequencing Reveal Two Genes Associated with Pollen Fertility in Tetraploid Rice
Source: Int J Mol Sci. 2021 Jan 15;22(2):841. doi: 10.3390/ijms22020841 (PMC7830325; doi:10.3390/ijms22020841)
Supplement: Supplementary file 1 [file ijms-22-00841-s001.zip › Supplementary Files/Additional File 1 2021-01-11.docx]

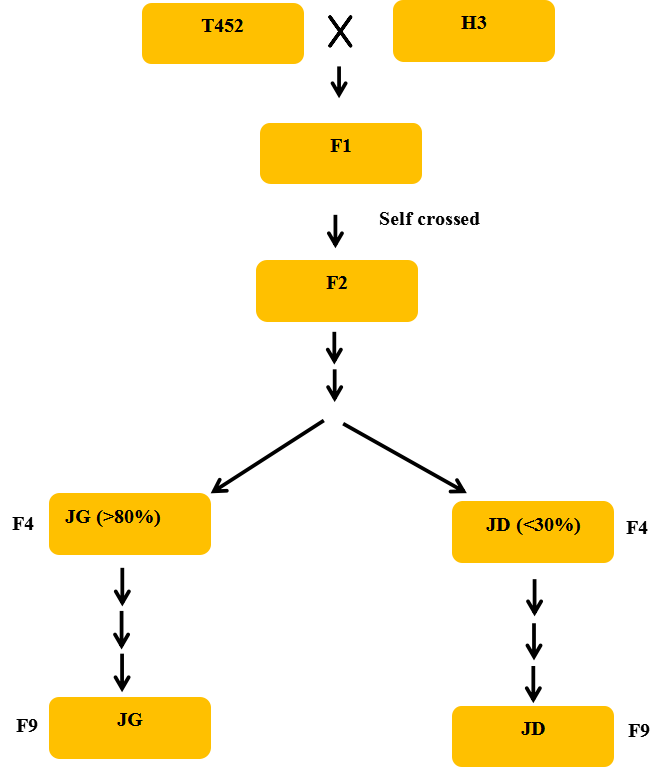


**Fig. S1** Breeding procedure of JG and JD inbred lines

Note: H3 represents the neo-tetraploid rice Huaduo 3; T452 represents autotetraploid rice; JG represents high fertility inbred line; JD represents low fertility inbred line.


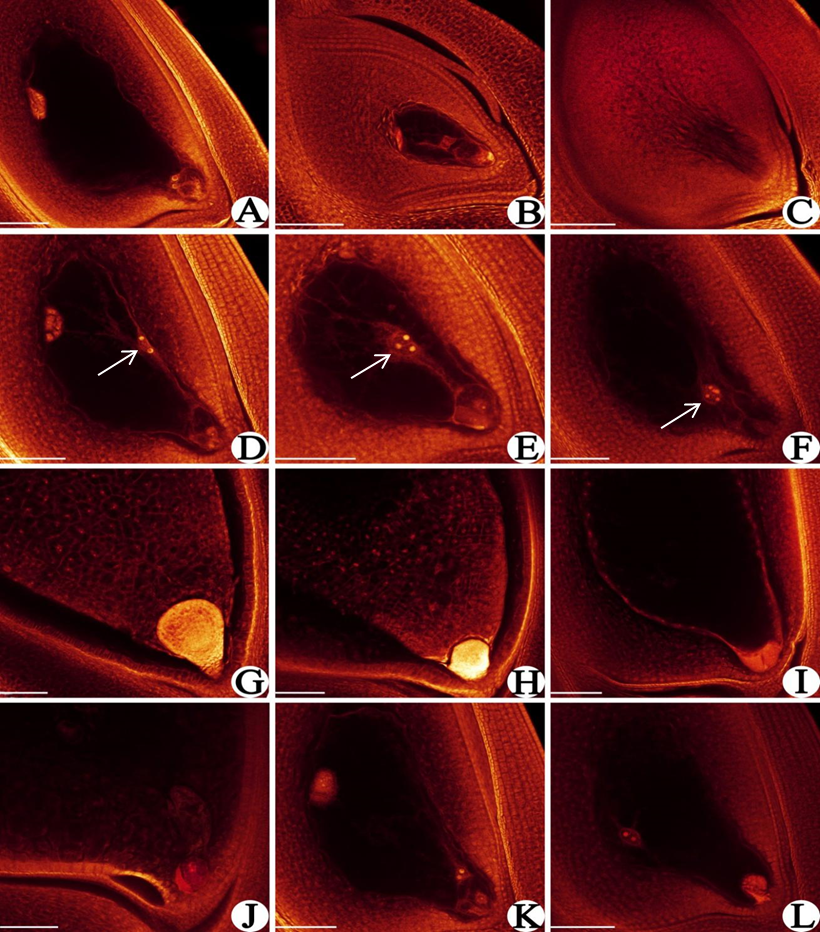


**Fig. S2** Mature embryo sac of neo-tetraploid inbred lines and their parents and embryo sac at 3 days after flowering (DAF)

(A to F) Mature embryo sac; (A) Normal embryo sac; (B) Small globular-shaped embryo sac; (C) Embryo sac degeneration; (D) Abnormal position of polar nuclei (arrow); (E) Three polar nuclei (arrow) in embryo sac; (F) Five polar nuclei (arrows); (G) 3 DAF, normal embryo; (H) 3 DAF, delayed embryo development; (I) 3 DAF, embryo and endosperm abnormality; (J) 3 DAF, inflated but unfertilized ovule; (K) 3 DAF, unfertilized embryo sac (L) 3 DAF, the egg cells are single fertilized. Bar = 100 μm.


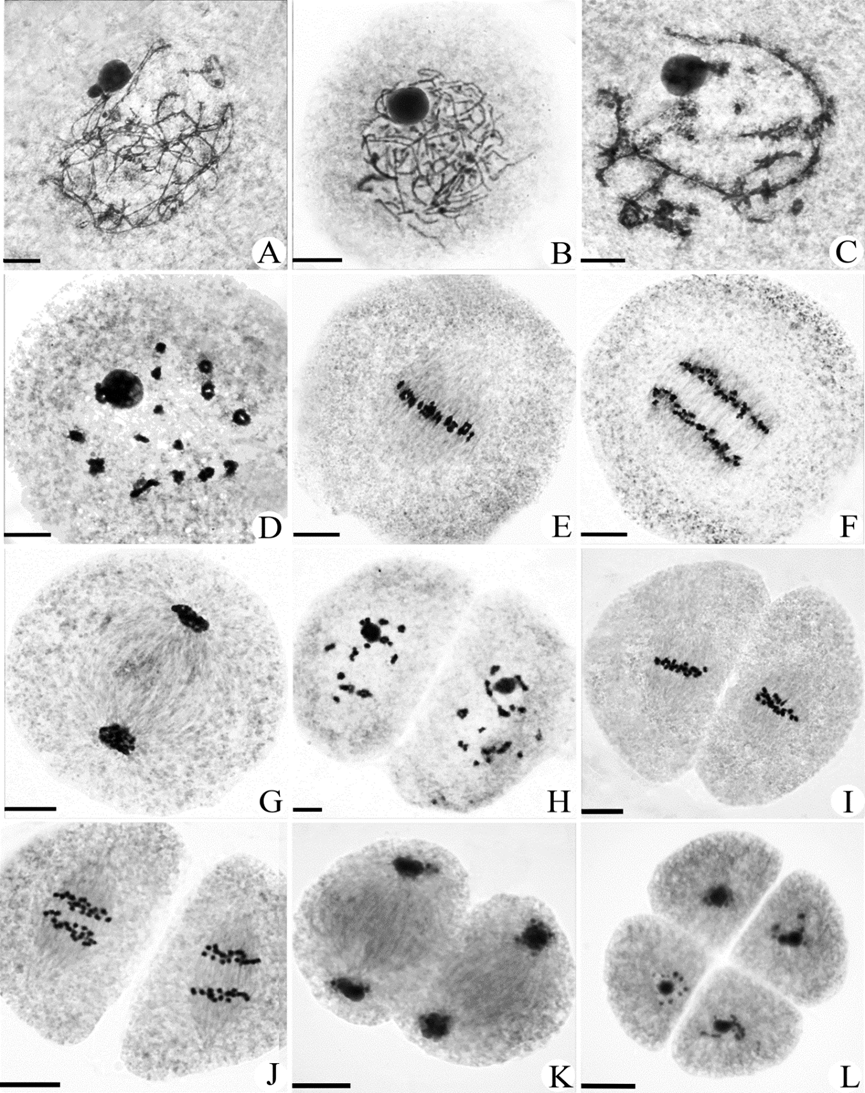


**Fig. S3** Normal chromosome behavior during pollen mother cells meiosis in JG.

A. zygotene; B. pachytene; C. diplotene; D. diakinesis; E. metaphase I; F. anaphase I; G. telophase I; H. prophase II; I. metaphase II; J. anaphase II; K. telophase II; L. tetrad. Bar = 10 μm.

**Table S1**. Pollen fertility of JG and JD hybrid lines and their parents

| Line | No. of pollen | Normal pollen (%) | Typical abortive  (%) | Empty pollen (%) | Stained abortive (%) | Small pollen (%) |
| --- | --- | --- | --- | --- | --- | --- |
| JG | 7221 | 88.53±4.79A | 7.31±0.97 | 3.09±0.00 | 2.87±0.00 | 0.73±0.00 |
| JD | 6566 | 25.08±5.09B | 18.44±2.65 | 14.85±2.10 | 55.17±6.32 | 1.23±0.00 |
| H3 | 5088 | 87.45±2.40A | 4.41±0.00 | 2.29±0.00 | 0.00±0.00 | 1.05±0.00 |
| T452 | 5065 | 23.70±6.75B | 7.80±1.48 | 6.17±0.99 | 54.48±4.77 | 2.95±0.74 |

Note: Capital letters A and B represent a significant difference at < 0.01

**Table S2** Quality Assessment of Whole Genome Sequencing in F_2_ Generation Mixed Pool

| Samples | Ave_depth | Cov_  ratio_  1× (%) | Cov_  ratio_  5× (%) | Cov_  ratio_  10× (%) | Mapped  (%) | Clean_  Reads | Clean_Base | Q30  (%) | GC  (%) |
| --- | --- | --- | --- | --- | --- | --- | --- | --- | --- |
| H3 | 25 | 94.84 | 91.83 | 88.40 | 98.59 | 38994632 | 11682834970 | 89.66 | 41.65 |
| T452 | 23 | 92.11 | 87.87 | 83.75 | 97.80 | 38366308 | 11492027086 | 89.35 | 42.21 |
| JG | 73 | 97.32 | 95.84 | 94.69 | 98.64 | 110638401 | 33145462092 | 91.79 | 41.58 |
| JD | 74 | 97.75 | 96.07 | 94.96 | 98.59 | 112518326 | 33707225852 | 91.51 | 42.34 |

**Table S3** Quality Assessment of whole genome sequencing of mixed pool in F_3_ Generation

| Samples | Ave_depth | Cov_  ratio_  1× (%) | Cov_  ratio_  5× (%) | Cov_  ratio_  10× (%) | Mapped  (%) | Clean_  Reads | Clean_Base | Q30  (%) | GC  (%) |
| --- | --- | --- | --- | --- | --- | --- | --- | --- | --- |
| H3 | 34 | 94.92 | 92.72 | 90.98 | 98.69 | 52344600 | 15670585998 | 90.88 | 43.16 |
| T452 | 28 | 91.86 | 87.95 | 84.86 | 98.44 | 41033642 | 12292580558 | 95.44 | 43.24 |
| JG | 67 | 97.28 | 95.74 | 94.62 | 98.63 | 99563070 | 29829986930 | 92.82 | 45.09 |
| JD | 67 | 98.08 | 96.19 | 94.69 | 98.01 | 98091904 | 29355540262 | 90.68 | 43.50 |

**Table S4** Quality evaluation of transcriptome sequencing in F_4_ generation pool

| Samples | Clean_Reads | Clean_Base | Q30 (%) | GC (%) |
| --- | --- | --- | --- | --- |
| H3 | 21285685 | 6366793114 | 94.12 | 56.23 |
| T452 | 20948785 | 6263753634 | 94.02 | 55.37 |
| JG | 154082424 | 45967108594 | 95.11 | 56.10 |
| JD | 104712475 | 31327731802 | 94.09 | 56.03 |
